# Detection of helminth ova genera using *in-situ* biosynthesis of gold nanoparticles

**DOI:** 10.1016/j.mex.2019.04.026

**Published:** 2019-04-30

**Authors:** Vivek B. Ravindran, Adam Truskewycz, Andrew S. Ball, Sarvesh K. Soni

**Affiliations:** School of Science, RMIT University, GPO Box 2476, Melbourne, VIC, 3001, Australia

**Keywords:** *In-situ* biosynthesis of metal nanoparticles by helminth ova, *Ascaris*, *Trichuris*, Wastewater, Helminth ova, Gold nanoparticles, SEM, EDX

## Abstract

In this study, a presumptive colorimetric method was used to detect and differentiate the ova of two major soil transmitted helminths in wastewater, *Ascaris* and *Trichuris*. Gold nanoparticles were synthesised following the reduction of tetrachloroauric acid by the surface moiety of *Ascaris suum*, resulting in a colour change. In contrast there was no colour change with *Trichuris suis* indicating the absence of gold nanoparticle synthesis. Analysis of the ova using scanning electron microscopy (SEM) revealed that the synthesis of nanoparticles on the surface of ova was confirmed as gold nanoparticles (91 w/w %) by energy dispersive X-ray analysis (EDX). This study indicated that the surface moieties of helminth ova could be a potential target for ova detection and differentiation using the biosynthesis of nanoparticles by colorimetry methods.

Three advantages:

• Simple colorimetry based method requiring no sophisticated devices.
• No trained personnel required.
• Cost-effective and can be a potential candidate for biosensors.

## Graphical abstract

**Figure:**
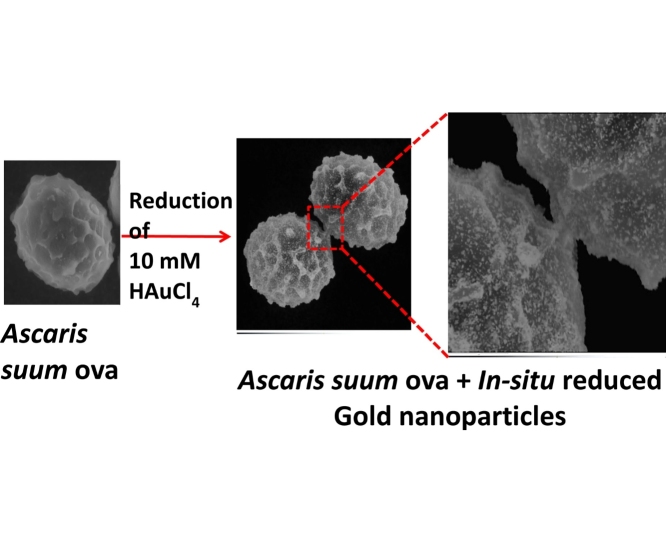

**Specification Table**

**Table:** 

Subject area:	Environmental Science
More specific subject area:	Detection of helminth ova
Method name:	*In-situ* biosynthesis of metal nanoparticles by helminth ova
Name and reference of the original method:	Ratul Kumar Das, Vinayak Laxman Pachapur, Linson Lonappan, Mitra Naghdi,Rama Pulicharla, Sampa Maiti, Maximiliano Cledon,Larios Martinez Araceli Dalila, Saurabh Jyoti Sarma, Satinder Kaur Brar (2017). Biological synthesis of metallic nanoparticles: plants, animals and microbial aspects. Nanotechnology for environmental engineering. Vol 2. P. 18

## Method details

*Ascaris lumbricoides* is a major soil-transmitted helminth (STH) with an estimated global prevalence of over a billion people, especially in South-east Asia, Latin America and Sub-Saharan Africa [1,2]. It is one of the most important causes of childhood malnutrition, cognitive impairment and gastrointestinal complaints. A low infective dose combined with environmental hardiness and excretion of up to 200,000 ova per day in the faeces of infected hosts enhances the infectivity of *Ascaris* [3]. The use of raw and partially treated wastewater for agriculture is considered one of the major epidemiological factors for the prevalence of *Ascaris* infections [4]. Several studies have shown a significant relationship between *Ascaris* infection and exposure to treated and untreated wastewater [5]. The ability of *A. lumbricoides* to survive wastewater treatment makes this an important pathogen to monitor as an indicator of effective water treatment and sanitation as recommended by the World Health Organisation (WHO). An upper limit of one helminth ova per litre is recommended for recycled water to be considered as suitable for unrestricted use [6].

Despite high standards of urban wastewater treatment and good sanitation systems in developed countries, the removal of *Ascaris* ova from wastewater remains crucial to allow the safe use of recycled water in agriculture [7]. Conventional monitoring for helminth ova in these environments relies on culture-based methods such as incubation and optical microscopy, which have many limitations, such as being time-consuming (up to 4 weeks) and laborious which hinder their usefulness as a detection tool [8]. Despite the advancement in molecular techniques, the need for sophisticated devices is a major constraint, especially in endemic and resource limited settings where reliable means of power supply may not be available [7].

These limitations have inspired the development of biosensors that could be used for rapid and sensitive detection of pathogens. While these biosensors have demonstrated significant advancements in subcellular characterisation, they are limited to use in a laboratory environment because they require specialised equipment and technical expertise [9]. There is a growing need for simple methods of detection that could be used at the point-of-care [10,11].

Gold nanoparticles (AuNPs) provide excellent platforms for the development of colorimetric biosensors as they can be easily functionalised displaying different colours depending on their size, shape and state of aggregation [12]. The conventional method for the synthesis of AuNPs involves the reduction of gold ions by reducing agents such as alkanethiols and alkylamines, or polymeric materials such as polyvinyl alcohol and gelatin to stabilise the AuNPs from aggregation [13]. However the use of capping agents for stabilisation and toxic chemicals for reductions may result in the formation of hazardous by-products, adversely affecting their use in diagnostics [14]. To overcome such limitations, various microbes [15] and biomacromolecules [16] have been used to synthesise AuNPs through redox reactions. In this study, helminth ova were differentiated based on their potential to synthesise gold nanoparticles *in-situ*. The ova shell of *Ascaris* species consists of i) a mucopolysaccharide uterine layer forming on the ova surface, typical irregularly thick structures of about 1.5 μm, ii) a highly structural chitin-protein layer with a thickness of 2 μm and iii) an inner lipid layer reaching a thickness of 0.8–1 μm which is an extremely resistant and impermeable osmotic barrier protecting the developing embryo against environments stress [17]. Consequently the difference in chitosan, lipid and protein in the ova shell will respond differently to the reduction of gold salt leading to the synthesis of gold nanoparticles. This strategy is used to colorimetrically distinguish the ova of two helminth genera, *Ascaris* and *Trichuris*. The synthesis of gold nanoparticles by the surface moieties of *Ascaris* ova was further analysed using scanning electron microscopy (SEM) and energy dispersive X-ray spectroscopy (EDX).

## Materials and methods

Tetrachloroauric acid (HAuCl_4_) was obtained from Merck (Australia) and prepared as 0.1 M using deionised MilliQ water. The ova of *Ascaris suum* (roundworm) and *Trichuris suis* (whipworm) utilised for this study were recovered from infected pig faecal samples that were obtained from Professor Rebecca Traub, Faculty of Veterinary and Agricultural Sciences, University of Melbourne (Australia). No animals were used for this study and the faecal samples were obtained solely for diagnostic purposes for a different study. Separation of ova from faecal samples (5 g) was performed by a simple flotation method with the addition of magnesium sulphate (specific gravity 1.20) in each 50 mL tube. The tubes were centrifuged twice at 800 *g* for 3 min after which the supernatant was passed through a 38 μM sieve and the material remaining on the sieve was washed with reverse osmosis water into a small beaker and transferred into 15 mL tubes and centrifuged at 800 *g* for a further 3 min. The supernatant was discarded and the ova that remained in the pellet were enumerated by optical microscopy (×200 magnification) using Whitlock Universal 4 chamber worm egg counting slides (J. A. Whitlock & Co, Australia), each of 500 μL capacity. The enumerated ova were aliquoted (1000 ± 20 ova) into 1 mL volume (5% potassium dichromate) in 2 mL Eppendorf tubes and stored at 4 °C. Ova (1000 ± 20) of each helminth were added onto 500 μL of 10 mM HAuCl_4_ and incubated at 37 °C for 24 h using a thermomixer at 650 rpm (Eppendorf, Australia). Control consisted of 500 μL of 10 mM HAuCl_4_ without helminth ova. The samples were visualised for colour change. Furthermore, the structure of helminth ova and the synthesis of AuNPs by the ova surface moiety were investigated by scanning electron microscope XL30 (Philips, Netherlands). The elemental composition of the nanoparticle colloids was evaluated using energy dispersive X-ray spectroscopy using an Oxford X-Max20 EDX Detector. The energy of the electron beam was maintained at 15 keV for both imaging and EDX analysis. SEM and EDX analysis was performed at the RMIT microscopy and microanalysis facility (RMMF), RMIT University, Australia. To confirm the lower limit of detection (LLOD) for visualization of colour change with the naked eye, helminth ova (1, 10, 50, 100, 200 and 500) were added onto 500 μL of 10 mM HAuCl_4_ and incubated at 37 °C for 24 h using a thermomixer (650 rpm).

## Results and discussion

**Fig. 1:**
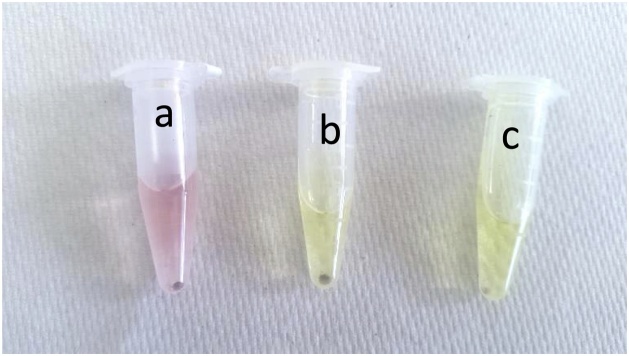
Simple colorimetry based differentiation of helminth ova: a) *A. suum* ova exhibiting colour change from yellow to ruby pink, b) *T. suis* ova showing no colour change and c) Control (without any ova).

The formation of AuNPs was indicated by the immediate colour change from light yellow to ruby pink in the Eppendorf tubes with *Ascaris* ova (Fig. 1). This colour change indicated the reduction of HAuCl_4_ leading to the formation of AuNPs. However no colour change was observed in the other helminth ova genera i.e. *Trichuris suis*.

**Fig. 2:**
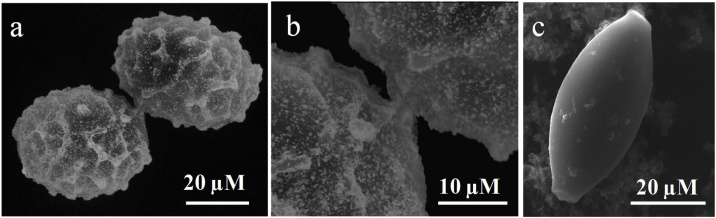
Imaging of helminth ova using Scanning Electron Microscopy: a) Presence of *in-situ* synthesized AuNPs on the surface of *A. suum* ova denoting the reduction of HAuCl_4_, b) high magnification image of 2a c) absence of AuNPs on *T. suis* ova surface.

SEM imaging showed the presence of AuNPs synthesized and bound to the surface moiety of *A. suum* ova (Fig. 2a and b). However, there were no NPs synthesized and attached to the surface moiety of *T. suis* ova (Fig. 2c).

**Fig. 3:**
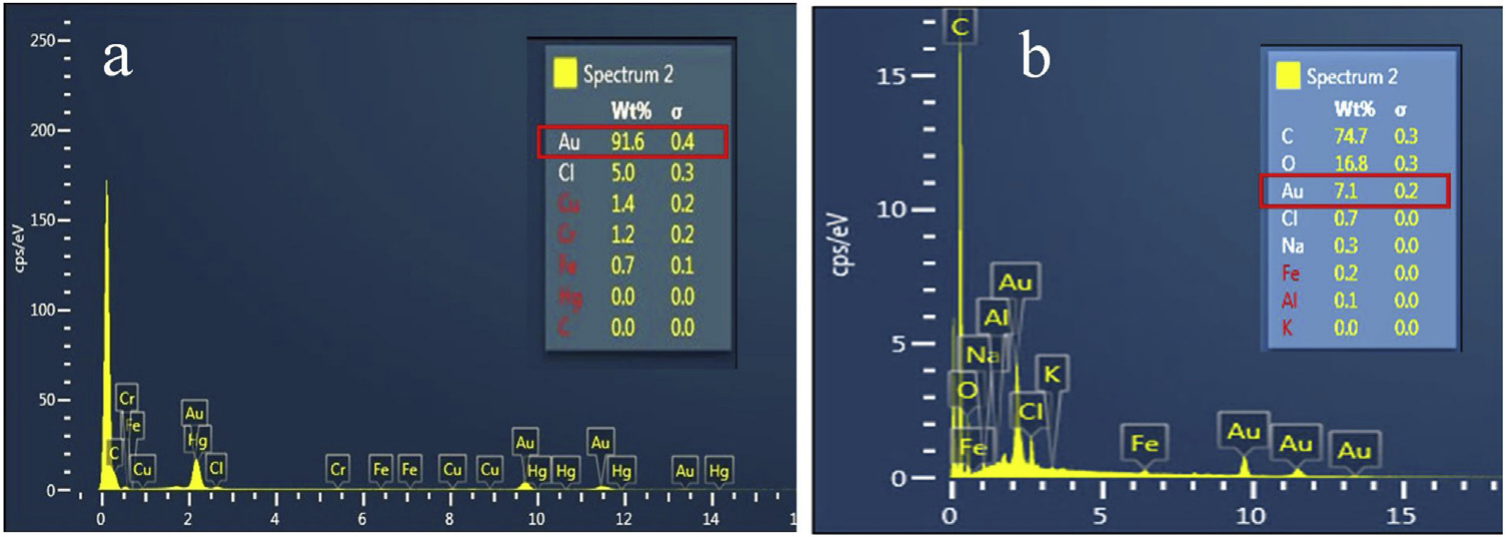
a) EDX analysis of selected area of *A. suum* ova showing 91 (w/w %) gold composition b) EDX analysis of selected area of *T. suis* ova showing only 7.1 (w/w %) gold composition.

EDX analysis of *A. suum* ova indicated that 91 w/w% of the surface moiety of *A. suum* ova was bound with gold nanoparticles (Fig. 3a) while there was significantly reduced synthesis of AuNPs (7.1% w/w) by *T. suis* ova (Fig. 3b).The differentiation in the synthesis of gold nanoparticles may be due to variations in ova shell structure between *Ascaris* and *Trichuris* ova. The outer surface consisting of mucopolysaccharides in *A. suum* is thicker than that in *T. suis* [18,19] which may be the reason for differential *in-situ* gold nanoparticle biosynthesis.

Furthermore, the sensitivity assay revealed that a colour change was observed between 50 and 500 helminth ova, however no colour change was visible with 1 and 10 helminth ova. Therefore the lower limit of detection was 50 helminth ova (Supplementary information: Fig S1) equivalent to 100 ova/L of wastewater (assuming a 50% ova recovery rate). However the assay has yet to be validated for the detection of STH ova in faecal samples allowing assessment of the potential application of this method for point-of-care testing.

## Conclusion

In this preliminary study, *A. suum* ova were selectively detected by a simple colorimetry method using *in-situ* metal biosynthesis of gold nanoparticles. This selective detection might have occurred due to the reduction of gold chloride by the interaction of surface moiety of *A. suum* ova and gold chloride thereby resulting in the synthesis of gold nanoparticles. Further analysis of the helminth ova using histochemical and chitosan/chitinase assay followed by X-ray diffraction and transmission electron microscopy (TEM) may determine the nanoparticle’s crystal phase leading to the differentiation of major soil transmitted helminths based on *in-situ* metal nanoparticle biosynthesis.
