# Supplementary material for: Detection of helminth ova genera using in-situ biosynthesis of gold nanoparticles
Source: MethodsX. 2019 Apr 30;6:993–7. doi: 10.1016/j.mex.2019.04.026 (PMC6506560; doi:10.1016/j.mex.2019.04.026)
Supplement: Supplementary file 1 [file mmc1.docx]

**Fig S1**


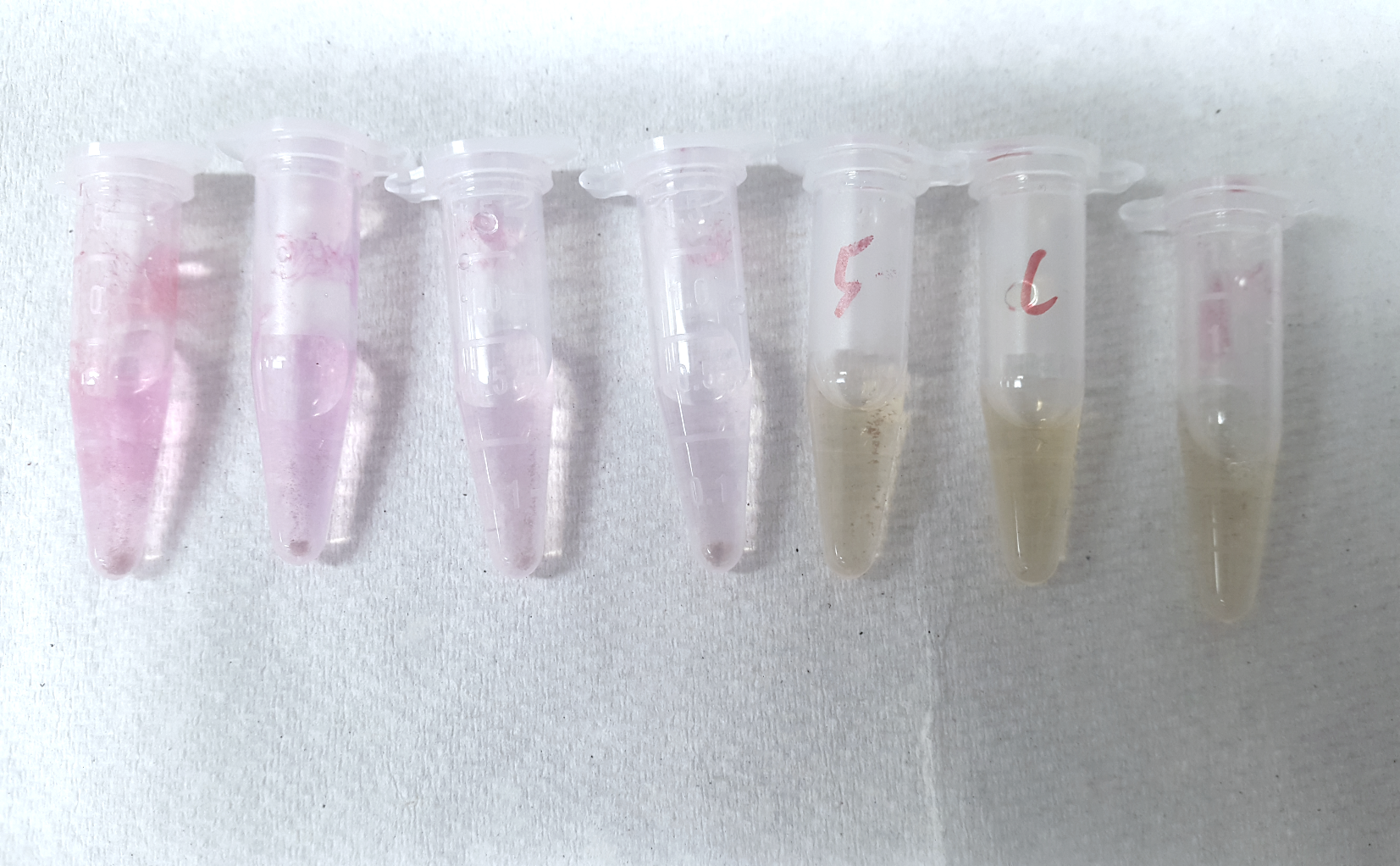


A B C D E F G

**Fig S1**: Sensitivity assay (limit of detection) for the detection of *A. suum* ova using colorimetric assay [A-500 ova, B-200, C-100, D-50, E-10, F-1, G- Control]
